# European Travel

**Published:** 1871-06

**Authors:** 


### EUROPEAN TRAVEL.

EVERY year it is becoming more common for Americans to take their children, daughters especially, abroad to be educated, and to be trained for family duties. The charges for the education of our girls at home are so enormous that many are forced, as a matter of economy, to seek a foreign education. At Manheim on the Rhine, three hundred dollars a year will pay all expenses for a girl. In this school all dress alike, the daughter of the titled and the commoner; this education includes that necessary for the wife and mother as well as for the drawing-room and reception. Nice, in France, has a delightful climate, where the Rev. N. C. Burt, D. D., of Cincinnati, Ohio, a man well known of all, has established a school for American girls.

